# A Protocol-Driven, Bedside Digital Conversational Agent to Support Nurse Teams and Mitigate Risks of Hospitalization in Older Adults: Case Control Pre-Post Study

**DOI:** 10.2196/13440

**Published:** 2019-10-17

**Authors:** Nicholas Bott, Sharon Wexler, Lin Drury, Chava Pollak, Victor Wang, Kathleen Scher, Sharon Narducci

**Affiliations:** 1 Clinical Excellence Research Center Department of Medicine Stanford University School of Medicine Stanford, CA United States; 2 Department of Psychology PGSP-Stanford Consortium Palo Alto, CA United States; 3 Pace University New York, NY United States; 4 Care.Coach Millbrae, CA United States; 5 Jamaica Hospital Medical Center New York, NY United States

**Keywords:** digital health, older adults, loneliness, delirium, falls, embodied conversational agent, chatbot, relational agent, information and communication technology

## Abstract

**Background:**

Hospitalized older adults often experience isolation and disorientation while receiving care, placing them at risk for many inpatient complications, including loneliness, depression, delirium, and falls. Embodied conversational agents (ECAs) are technological entities that can interact with people through spoken conversation. Some ECAs are also relational agents, which build and maintain socioemotional relationships with people across multiple interactions. This study utilized a novel form of relational ECA, provided by Care Coach (care.coach, inc): an animated animal avatar on a tablet device, monitored and controlled by live health advocates. The ECA implemented algorithm-based clinical protocols for hospitalized older adults, such as reorienting patients to mitigate delirium risk, eliciting toileting needs to prevent falls, and engaging patients in social interaction to facilitate social engagement. Previous pilot studies of the Care Coach avatar have demonstrated the ECA’s usability and efficacy in home-dwelling older adults. Further study among hospitalized older adults in a larger experimental trial is needed to demonstrate its effectiveness.

**Objective:**

The aim of the study was to examine the effect of a human-in-the-loop, protocol-driven relational ECA on loneliness, depression, delirium, and falls among diverse hospitalized older adults.

**Methods:**

This was a clinical trial of 95 adults over the age of 65 years, hospitalized at an inner-city community hospital. Intervention participants received an avatar for the duration of their hospital stay; participants on a control unit received a daily 15-min visit from a nursing student. Measures of loneliness (3-item University of California, Los Angeles Loneliness Scale), depression (15-item Geriatric Depression Scale), and delirium (confusion assessment method) were administered upon study enrollment and before discharge.

**Results:**

Participants who received the avatar during hospitalization had lower frequency of delirium at discharge (*P*<.001), reported fewer symptoms of loneliness (*P*=.01), and experienced fewer falls than control participants. There were no significant differences in self-reported depressive symptoms.

**Conclusions:**

The study findings validate the use of human-in-the-loop, relational ECAs among diverse hospitalized older adults.

## Introduction

### Background

In 2014, 19% of Medicare beneficiaries had at least one inpatient stay covered under Medicare Part A [1]. In addition to the illness or injury requiring acute care, the unfamiliar and stressful environment of the hospital increases risk of loneliness, depression, delirium, and falls in these patients. Loneliness is a frequent occurrence in older adults, and it is a documented predictor of poor health. One in 3 older adults reports loneliness in the United States [2], and a recent American Association of Retired Persons (AARP) survey found that approximately 42.6 million older adults suffer from chronic loneliness [3]; research demonstrates that loneliness is linked to a variety of negative health outcomes, including high blood pressure [4], disability [5], functional decline [2], depression [6], and cognitive decline [7]. These comorbidities may consequently increase the need for health care, and these are linked to greater health care utilization [8].

For older adults, the hospital setting is also a precipitant for symptoms of depression and loss of control [9]. Depression, in turn, may stymie recovery and increase the length of hospital stays [10]. At the same time, a recent international study found that informal psychological support was inversely related with depressive symptoms in hospitalized older adults [10]. The challenge remains to find ways to incorporate low-cost interventions that provide psychological support within the fast-paced hospital setting.

In addition to loneliness and depression, delirium and falls represent adverse events for hospitalized older adults. Delirium is a serious but preventable condition associated with morbidity and mortality, occurring as frequently as 50% of all hospitalized older adults [11]. Owing to its limited recognition within inpatient settings, the presence of delirium is associated with inappropriate use of sedation, sitters, and restraints [12]. Furthermore, delirium contributes to falls, cognitive decline, disability, morbidity, and mortality, with estimated direct health care costs over US $164 billion a year in the United States [11,13,14].

Falls are a common adverse event associated with hospitalization, and fall risk increases with age. Delirium significantly increases the risk of falls in the hospital setting [11,13,14]. In the United States, all of these risks are greatest among patients with limited English comprehension, low health literacy, and socioeconomic disadvantage [15]. Evidence demonstrates that an individualized multifactorial approach to fall prevention, including alert wristbands, room signage, staff and patient education, footwear, toileting schedules, exercise, and movement alarms, is effective [16]. For patients with cognitive impairment, increased nursing surveillance is also an effective intervention [17].

In view of the relationship between these adverse events and morbidity, as well as decreased quality of life, increased health care usage, and subsequent cost, it is imperative to find interventions that can reduce the risk of these outcomes in hospitalized older adults. The Hospital Elder Life Program (HELP) is a multidomain nonpharmacological intervention that has been shown to be effective in mitigating the risk of these adverse events. A meta-analysis of 14 interventional studies demonstrated efficacy in reducing incidence of delirium [18], falls [18], and functional status [18]. Separately, HELP has also been shown to improve symptoms of patient loneliness [19]. HELP volunteers deliver protocols targeting multiple risk factors, including orientation, mobilization, vision, hearing, hydration, nutrition, and sleep. HELP is used in more than 200 hospitals worldwide, and it serves as the gold-standard nonpharmacological intervention for the risk mitigation and management of delirium, cognitive and functional decline, falls, and 1:1 observation among older adults [18-20]. Although the HELP program is cost effective, it requires substantial training and monitoring of a large team of volunteers, which can serve as a barrier to adoption, especially in safety-net facilities with limited resources, serving high-risk patient populations [21-23]. To date, research has not determined whether human-driven virtual companionship can approximate the outcomes of physical volunteers for hospitalized older adults.

The health technology literature is abundant and continues to grow; nevertheless, there is a dearth of information on the use of technology as a social relational agent for older adults. Embodied conversational agents (ECAs) are technological entities that can interact with people through spoken conversation. Some ECAs also function as relational agents and are designed to leverage these conversational interactions to build and maintain social-emotional relationships with people [24]. The Care Coach avatar is a relational ECA that interacts with patients primarily through speech, along with its own visual appearance, digital pictures, and audio or music that can play through the tablet speaker in the same way as a HELP volunteer would in vivo. The Care Coach avatar is powered by a human-in-the-loop software system, so-called because of a round-the-clock team of live health advocates monitoring and controlling each avatar. As a result, avatar conversations with patients and care teams are conducted through natural speech, allowing complex patients with cognitive and functional impairments to be engaged effectively, regardless of their technical abilities or inclination. Severely hearing-impaired patients may even understand the avatar by reading the captions displayed above the avatar’s head on the tablet screen.

Existing studies have generally evaluated the ease of use and acceptability of health technology, and they have specifically evaluated social agents as a tool for decreasing loneliness and social isolation in older adults [25-29]. Khosravi et al conducted a systematic review to synthesize studies investigating the role of technology in addressing loneliness and social isolation in older adults [29]. These studies included different technological modalities offering various means of engagement, including computer/internet use, robotics, video games, personal reminder information and social management systems, social networking sites, tele-care, and 3D virtual environments. Efficacy data included reduced loneliness, development and maintenance of social relationships, increased independent living, decreased social isolation, companionship, cognitive stimulation, and entertainment [29].

In a pilot study of home-dwelling older adults, Chi et al provided a qualitative perspective on utility and comfort with the Care Coach avatar presented in this paper [26]. Participants reported positive results in terms of companionship, social support, and health information. However, some concerns included internet connectivity, privacy, and cost. In addition, some participants reported variations in the quality of the avatar’s conversations, associated with differing staff members operating the avatar, highlighting the importance of the human aspect of this technological experience. Demiris et al conducted a study utilizing the Care Coach platform in home-dwelling older adults with mild cognitive impairment and reported improvements in cognition and social support, along with decreases in depressive symptoms [30].

### Objectives

The results of the previous pilot study demonstrated the feasibility of the Care Coach platform to improve clinically meaningful aspects of patient care in the home. Given the additional risks of delirium and falls, in addition to loneliness and depression, that hospitalized older adults experience during hospitalization, a less resource-intensive deployment of the multidomain interventions, included within the HELP program through a relational ECA, may provide a meaningful and scalable method for mitigating adverse events. This study investigated the efficacy of a human-in-the-loop, protocol-driven relational ECA, compared with control participants among hospitalized older adults on self-reported measures of loneliness, depression, and clinician-reported occurrence of delirium and falls. We hypothesized that the use of such an intervention would decrease self-reported loneliness and depression, as well as reduce the occurrence of delirium and falls during hospitalization.

## Methods

### Experimental Intervention

**Figure 1:**
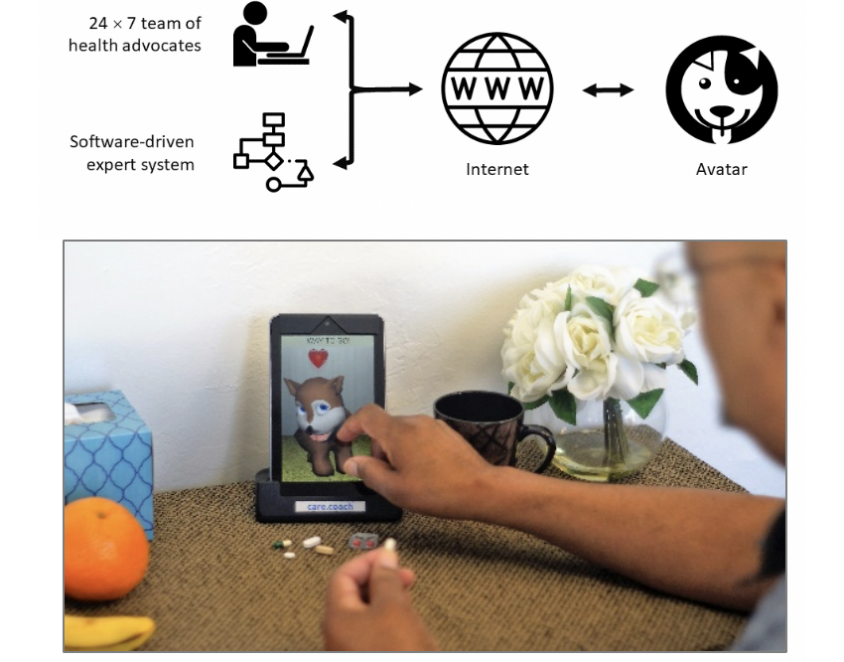
The Care Coach avatar system design (top). An avatar encourages a patient to take his medications (bottom).

The relational ECA software platform utilized in the study was provided by Care Coach, a private company based in California, and it comprises an internet-based communication system, designed to provide 24-hour psychosocial and health care support for patients through an integrative, person-centered approach [31]. A relational ECA serves as the patient-facing interface, appearing as an animated dog or cat on the display screen of a tablet computing device (see Figure 1). The digital animal responds to touch and demonstrates emotions appropriate to the conversation or other interaction state, including facial expressions, bodily reaction to touch and petting, heart symbols, tears, sleeping, and snoring. The Care Coach platform represents a real-time fusion of human and software intelligence, powered by a team of live health advocates who see, hear, and speak with each patient through the avatar of the digital animal. Health advocates are available 24 hours a day, and they are guided by software algorithms to implement clinical protocols. The use of a nonhuman avatar plays several important roles. First, previous studies have found unique clinical benefits associated with nonhuman avatar relational agents [32]. Second, an avatar relational agent provides greater continuity of care across the duration of the hospital stay, avoiding coverage gaps and challenges with establishing and re-establishing a care relationship throughout the duration of the hospital stay. Finally, the use of a nonhuman avatar provides a clear distinction in role for patients. The avatar relational agent is not a medical professional that provides clinical information, and the use of a nonhuman avatar (as opposed to a nurse of clinician avatar) visually clarifies this distinction.

**Figure 2:**
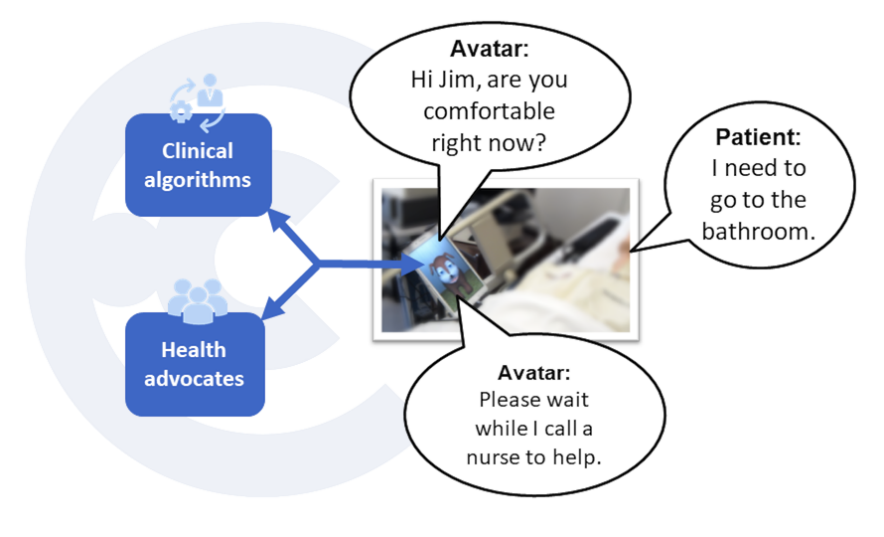
An example of a simple fall prevention protocol.

Using a proprietary Web-based work interface, health advocates can sequentially monitor and engage 12 or more patients, and they can simultaneously monitor and engage up to 2 patients. Health advocates observe and listen through the audiovisual feed from the avatar device, communicating in real time with each patient by sending text commands, which are converted into the avatar’s voice using a text-to-speech engine. Thus, the health advocates contribute their human abilities for natural language understanding, in both English and Spanish, and sociable, compassionate, and conversational responses to help patients build personal relationships with the avatar. Their human abilities are augmented through a software-driven system embedded into the work interface, which uses branching logic and prescripted conversational content to guide health advocates through evidence-based intervention protocols, such as cognitive engagement or assessment, reorientation, toileting checks, and fall prevention (see Figure 2), alerting the nurse station on the hospital unit by phone of any issues potentially requiring immediate action.

In this study, the avatars were programmed with specific protocols, repeating for each patient every 2 days (see Multimedia Appendix 1). For example, the Care Coach system was programmed to proactively ask about comfort and need of bathroom use, seeking assistance on behalf of the patient by calling the nursing station. Studies show that approximately half of all falls in the hospital occur either in the bathroom or on the way to the bathroom [33,34]. In addition to scheduled protocols, each avatar provided engagement on an unscheduled, informal basis through relationship-building conversations about participant interests, family, and news, as well as through showing pictures, playing music, guided meditation/relaxation tracks, and engaging the patient in brain games.

As a relational ECA, the health advocate is able to wake up the avatar with an accompanying *wake noise* whenever the audiovisual stream is started and visually put the avatar to sleep whenever the audiovisual stream stops. As the Care Coach platform utilizes a human-in-the-loop health advocate, all patient information is treated as protected health information, and all health advocates receive Health Insurance Portability and Accountability Act training. Moreover, patients may ask the avatar for privacy at any time, as long as such a request is consistent with the safety monitoring requirements of the health care provider or proxy.

### Study Design

This clinical study used a case control quasi-experimental pre-post design. Participants were recruited from 3 medical-surgical units in a 600+ bed community hospital. Units were similar in size (34-37 beds), average daily census (32 patients), and average length of stay (3-6 days). One unit served as the control unit, with 2 units serving as intervention units. Patients were enrolled and followed by a team of research assistants (RAs), comprising undergraduate and graduate nursing and computer science students. The RAs were trained, and they followed a structured protocol and scripts. Project management, as well as scheduling of RAs, was conducted by a graduate nursing student.

### Setting

The study was conducted in an urban community hospital in New York City, which targets the underserved. The hospital is in the borough of Queens, one of the most ethnically diverse urban areas in the world [35]. The majority of the borough’s population identifies as nonwhite, with strong representation from black, Asian, and Hispanic races [36]. Per capita income is US $28,814, with 19% of adults reporting less than high school education, and 11% of adults reporting lack of health insurance [36]. The hospital has 408 beds and approximately 120,000 emergency department visits annually [37].

### Participants and Procedures

Initial screening of potential participants was conducted by the nurse managers of the respective nursing units during daily rounds. Inclusion criteria were patients over the age of 65, admitted to 1 of the study units, who could give informed consent or had a proxy who could provide consent. Exclusion criteria were patients who could not provide informed consent with no proxy, as well as patients who wandered, demonstrated aggressive combative behavior with intent to harm self or others, or were experiencing alcohol or drug withdrawal. Patients who were noncommunicative or did not speak English were also excluded from the study. Eligible patients were referred to an RA. RAs discussed the study, obtained informed consent, and administered the enrollment instruments, which comprised a demographic questionnaire and assessment instruments (detailed below). The enrollment visit lasted approximately 15 min per participant.

A total of 2 units were utilized for the experimental intervention, with a third unit utilized as a control unit. All patients recruited from either of the intervention units were enrolled into the intervention group. All patients recruited from the control unit were enrolled into the control group. Owing to the pace of recruitment, enrollment in the second experimental unit began approximately 45 days after the first experimental unit. Enrolled patients on the intervention units selected either a dog or a cat avatar. RAs demonstrated how the avatar works and verified that each subject was comfortable with the device before leaving the bedside. RAs visited each subject daily throughout the hospitalization to confirm that each avatar was functioning and within view of the patient. Upon 24-hour discharge notification, RAs administered the postassessment instruments. Subjects discharged home or to another facility had the option of taking their avatar with them. Home-based data were collected on those participants utilizing the avatar postdischarge, which will be the focus of a separate follow-up study. Relational agent theory [38] suggests that conversational variety is essential to sustaining interaction between the subject and the avatar. Extending the period over which a protocol set is repeated promotes sustained engagement; therefore, rather than daily repetition, a 2-day rotation period was designed on the basis of an expected patient length of stay of approximately 4 days. Enrolled patients on the control unit received the same preassessments as the intervention participants. RAs visited each control participant daily throughout the hospitalization to provide a “dose” of RA contact, similar to that received by the intervention participants. RAs used a rounding script (see Multimedia Appendix 2) for both control and intervention patients, to ensure consistency among visits and equalize the influence of human contact on the outcomes being measured. Upon 24-hour discharge notification, RAs administered postassessment instruments to the control patients. Throughout the study, RAs recorded observations on the subjects’ responses to the avatars, using the observation checklist. All procedures were approved by the Jamaica Hospital and PACE University Institutional Review Boards.

### Measures

Demographic variables, including age, gender, education, race/ethnicity, and living arrangements, were collected upon enrollment. Discharge destination and living arrangements were collected up to 24 hours before discharge. Delirium, loneliness, and depression were assessed using the instruments detailed below. All instruments were administered at the time of enrollment into the study and upon discharge.

#### Delirium

The confusion assessment method (CAM) was utilized to screen for delirium [39]. The CAM includes an instrument and diagnostic algorithm for identification of delirium, assessing the presence, severity, and fluctuation of 9 delirium features, including the following: acute onset, inattention, disorganized thinking, altered level of consciousness, disorientation, memory impairment, perceptual disturbances, psychomotor agitation or retardation, and altered sleep-wake cycle. The CAM diagnostic algorithm is based on 4 cardinal features of delirium: (1) acute onset and fluctuating course, (2) inattention, (3) disorganized thinking, and (4) altered level of consciousness. A diagnosis of delirium utilizing the CAM requires the presence of features 1, 2, and either 3 or 4. The CAM has been used extensively for assessment of delirium among hospitalized older adults, and it has demonstrated excellent sensitivity and specificity among large and small samples of older adults [39].

#### Loneliness

Loneliness was measured using the University of California Los Angeles (UCLA) Loneliness Scale Short Form [40]. Participants rated themselves on a scale of “1 to 3” on the 3 items (“hardly ever”=1, “some of the time”=2, and “often”=3), with a total score ranging from 3 to 9. Higher scores indicate more loneliness. The short form has demonstrated reliability with the long form of the measure [40].

#### Depression

Depression was measured using the Geriatric Depression Scale (GDS) 15-item short form [41]. Of the 15 items, 10 indicate the presence of depression when answered positively, whereas the rest indicate depression when answered negatively. Scores from 0 to 4 are considered normal, depending on age, education, and complaints; scores from 5 to 8 indicate mild depression, 9 to 11 indicate moderate depression, and 12 to 15 indicate severe depression. The GDS has been used extensively to measure depressive symptoms among older adults [42].

#### Falls

Data on falls were obtained from existing unit-based quality improvement data from the hospital. Incidence data are recorded quarterly in hospital reports and presented as a ratio in the form of falls per 1000 patient days [43]. The fall incidence data for this study were collected over 2 full calendar quarters, comprising the 3-month period, preceding any intervention, and the following 3-month period, during which the intervention was applied. The control unit did not receive any avatars in either 3-month period. One intervention unit received avatars and had all its enrolled patients treated as subjects during the 3-month intervention period. Owing to recruitment cadence, the additional intervention unit started the 3-month intervention period approximately 45 days into the calendar quarter. As a result, the falls data on the second intervention unit represent a mixed unit, with the first half of the quarterly falls data not including participants receiving the intervention. Only summary data on quarterly fall rates by unit and time period were available. As a result, these data were interpreted in relationship to baseline data and in relationship to the national average [44] (see Multimedia Appendix 3).

### Power Analysis

Estimated sample size for this study utilized published results of the CAM, UCLA 3-item loneliness scale, and GDS. Medium-to-large effect sizes have been observed in studies utilizing the CAM, UCLA 3-item loneliness scale, and the GDS among hospitalized older adults [45,46]. Sample size calculation utilized an expected medium effect size (Cohen *d*=0.60), with alpha set at .05, and assumed power of 80% for analysis of variance yielded an expected sample size of 90. The study sample is powered at greater than 80% to detect a medium effect between groups.

### Data Analysis

Two-tailed Student *t* test and chi-square cross tabulations were used for descriptive analysis of participants. McNemar test was utilized to compare change in frequency of delirium within intervention and control groups. Analysis of covariance was used to analyze the differences among intervention and control group means on measures of loneliness and depression. Sex, baseline self-reported loneliness, and baseline MiniCog score were included as covariates in the loneliness analysis. Sex, baseline self-reported depressive symptoms, and baseline MiniCog score were included in the depression analysis. Quarterly fall rates for intervention and control units were examined descriptively in relationship to baseline data and to the national average. The analyses for this study were done using the SPSS version 25 (SPSS Inc).

## Results

### Descriptive Analysis

A total of 95 participants were included in the analyses (intervention group n=41; control group n=54). There were no differences between the control and intervention groups on age, race, place of residence, discharge location, or language (*P*>.05). The intervention group (68%, 28/41 female) included more female participants than the control group (44%, 24/54 female; *P*=.02). Across groups, participants were predominantly female, English-speaking African Americans with a mean age of 76 years. They resided at home and were discharged to home post hospital stay (Table 1).

On average, the avatars checked in with participants in the intervention group (a health advocate started the audio/video stream, visually waking up the avatar) 71.3 times per day per patient. Avatars engaged intervention participants for 61 min per day (including average use of 11.5 images or audio files) and completed, on average, 6.5 protocol-driven tasks per day (Table 2).

### Delirium

The presence of delirium at enrollment and discharge was analyzed within each group. McNemar tests found a significant reduction in delirium presence from enrollment to discharge in the intervention group (*P*<.001). There was no change in frequency of delirium presence within the control group (*P*=.25; Table 3).

### Loneliness

A general linear model examining admission and discharge endorsement on the UCLA Loneliness Scale indicated that participants with avatars experienced a decrease in loneliness (*P*=.01) compared with participants in the control group (see Table 3).

### Depression

A general linear model examining admission and discharge endorsement of depression symptomology on the GDS between groups showed no statistically significant difference in depression between participants with and without avatars (see Table 3).

### Falls

Quarterly unit-based quality improvement data on incidence of falls per 1000 patient days were examined across each of the 3 units. Falls rate was reduced by 33% on the avatar unit, with partial data collection. Falls rate was reduced by 82% on the avatar unit, with complete data collection. Frequency of falls increased on the control unit by 86%.

**Table 1:** Patient characteristics by study group.

Characteristics		Intervention (n=41)	Control (n=54)	*P* value
Age (years), mean (SD)		76.88 (8.85)	76.22 (8.05)	.70
**Race, n (%)**				.68
	White	10 (24)	14 (26)	
	African American	22 (54)	22 (41)	
	Asian/Pacific Islander	5 (12)	12 (22)	
	Hispanic	3 (7)	5 (9)	
	Other	1 (2)	1 (2)	
**Sex, n (%)**				.02^a^
	Male	13 (32)	30 (56)	
	Female	28 (68)	24 (44)	
**Residence before admission, n (%)**				.16
	Home	38 (93)	46 (85)	
	Nursing home	2 (5)	8 (15)	
	Other	1 (2)	0 (0)	
**Discharge location, n (%)**				.59
	Homeless	1 (2)	0 (0)	
	Home	32 (78)	45 (83)	
	Nursing home	5 (12)	7 (13)	
	Short-term rehabilitation	1 (3)	0 (0)	
	Other	2 (5)	2 (4)	
**English as a second language, n (%)**				.34
	No	25 (61)	28 (52)	
	Yes	16 (39)	26 (48)	

^a^*P*<.05.

**Table 2:** Patient engagement data (n=41).

Engagement metric	Mean (SD) per day
Number of check-ins	71.30 (7.46)
Observational and engagement time (min)	61.00 (40.61)
Media files used	11.50 (9.04)
Protocol tasks completed	6.5 (6.03)

**Table 3:** Outcome results.

Outcome variable	Intervention pre (n=41)	Intervention post (n=41)	Control pre (n=54)	Control post (n=54)	*P* value	Partial eta squared
Delirium, n/N (%)	12/29 (41)	1/40 (3)	6/48 (13)	3/51 (6)	<.001/.25	—^a^
Loneliness, mean (SD)	4.98 (2.17)	3.76 (1.53)	4.72 (1.74)	4.35 (1.70)	.01	0.07
Depression, mean (SD)	4.2 (3.2)	4.02 (2.99)	4.19 (3.5)	3.87 (2.99)	.46	0.006

^a^Not applicable.

## Discussion

### Principal Findings

This study investigated the efficacy of a human-in-the-loop, relational ECA in the form of a tablet-based virtual service animal avatar. Measures of delirium, loneliness, depression, and falls were investigated in a case-control study of 95 hospitalized adults over the age of 65 at an inner-city community hospital. Analysis of admission and discharge data indicated that intervention participants experienced lower frequency of delirium at discharge (*P*<.001) and a reduction in symptoms of loneliness (*P*=.01). Quarterly unit falls per 1000 patient days indicated that falls on the control unit increased by 86%. Falls on the intervention unit with delayed data collection were reduced by 33%. Falls on the intervention unit with complete data collection were reduced by 82%. There were no differences between groups in self-reported depressive symptoms. These results are consistent with previous research investigating devices that are used for a variety of needs specific to older adults, including safety and fall prevention, assistance with physical tasks that support activities of daily living, prevention of hospitalization, and social connectedness [28,29].

The avatar intervention resulted in a 91% reduction in delirium at discharge, with 11 participants meeting criteria for delirium with the CAM upon admission and 1 meeting criteria for delirium upon discharge. Although no individuals in either the control or intervention units acquired delirium after beginning the study, the Care Coach intervention’s ability to help resolve delirium within a short period of days is comparable with data reported on HELP intervention among hospitalized older adults [21].

With respect to falls, the reduction observed across intervention units is likely attributable to the nature of preprogrammed avatar protocols assisting with nursing calls to assist with mobility and toileting. The reduction in falls seen across the study duration provides further validation for the use of a human-in-the-loop, tablet-based intervention to provide a means for hospitalized older adults to communicate with health care providers. This is important, given the role that alarm fatigue can play in health care service delivery [47]. The reason for the increase in falls rate on the control unit is not known.

There are currently 12 million Americans over the age 65 years living alone [48]. In a survey published by investigating the frequency of loneliness among 3000 older adults, 35% were categorized as being lonely [49]. The effect of the avatar intervention on symptoms of loneliness suggests that this form of human-in-the-loop virtual engagement is an effective means for combating this issue while adults are hospitalized. This is noteworthy, given that the participants without avatars received personal visits from RAs for approximately 15 min each hospital day. Contrary to our hypothesis, the avatar intervention did not demonstrate reduction in depressive symptoms when compared with the control group. This may be attributable to the tenacity of depression among older adults and the relatively short exposure to the avatars associated with the hospital stay (averaging 4 days). It may also be because of the relatively minimal number of depressive symptoms endorsed by participants across both groups at baseline.

An important implication of this study is the feasibility of larger deployment. As a low-cost and sustainable intervention, the marginal resources required to deploy such a human-in-the-loop avatar intervention model would comprise marginal staffing effort and minimal technology components, including health advocate staffing (which can take advantage of an abundant remote workforce), certified nursing assistant’s effort to setup/sanitize the device (<US $1/patient day), wear and tear on the tablet device and related hardware (< US $1/patient day), and internet connection and server costs (approximately US $1/patient day on cellular data or approximately US $0/patient day with a reliable Wi-Fi network). Furthermore, participant learning curve is minimal, as the Care Coach technology platform does not require previous information technology fluency or use. Participants with mobility issues can engage with this technology, as the patient only needs to talk with the avatar, with no manual effort required. The digital animal avatar also solves such issues as the cost of caring for pets and potential animal-related allergies. Finally, preliminary evidence presented in this paper suggests the avatar may represent a superior intervention for risk reduction of delirium and falls over that of a patient sitter. Current evidence does not support the efficacy of patient sitters for reducing falls, and although there is a lack of evidence regarding patient sitters and delirium, a passive clinical care member in a patient’s room would likely have minimal impact on delirium risk.

Additional research is needed to establish the long-term effects of an avatar on the well-being of patients and their ability to provide self-care. Patients who utilized the avatar during this study experienced less delirium and fell less than those patients who did not utilize the avatar. Given that both delirium and falls have significant morbidity and mortality in older adults and represent a major financial burden to the health care system, the use of this technology could potentially improve outcomes for hospitalized older adults. The cost of this intervention is significantly less than the cost of a fall (the average hospital cost for a fall injury is over US $30,000) [50] or the potential cost of increased length of stay because of delirium, which is an average increase of 7.78 days per case of incident delirium [51].

### Limitations

This clinical study was limited by several factors. Although the sample size was sufficient, the intervention arm included convenience sampling and a potential bias in the referral process by the nurse managers. Given the deployment of this intervention in a real-world point-of-care environment, potential differences in unit care model and participant exposure to care across units could not be controlled. In addition, only patients who were able to understand and speak English were enrolled. In a very ethnically diverse setting, with many non-English speaking patients, many potential participants were excluded. However, the study location and population provided for the recruitment of a diverse population. Falls data were only available quarterly by unit. This limited investigation of falls data to percentage changes within units. The slower recruitment cadence on the second experimental unit in combination with quarterly falls data resulted in partial data on falls for this unit. Finally, more granular data on individual patients were not available, which prevented investigation into potential effects related to specific patient medical comorbidities.

### Conclusions

In conclusion, this study provides additional quantitative support to the clinical efficacy of the Care Coach avatar platform for hospitalized older adults. As a human-in-the-loop, tablet-based intervention, this technology provides a novel, scalable solution to mitigate the risks of delirium, loneliness, and falls among diverse hospitalized older adults. Future study of the Care Coach platform, including a randomized control trial (NCT03832192), will allow the replicability of these findings to be tested in a well-characterized and randomized patient population.

## Appendix

Multimedia Appendix 1 Avatar protocols.

Multimedia Appendix 2 Rounding script.

Multimedia Appendix 3 Quarterly fall rates by hospital unit and time periods.

## Abbreviations

CAM: confusion assessment method
ECA: embodied conversational agent
GDS: Geriatric Depression Scale
HELP: The Hospital Elder Life Program
RA: research assistant
UCLA: University of California Los Angeles
