# Supplementary material for: A Protocol-Driven, Bedside Digital Conversational Agent to Support Nurse Teams and Mitigate Risks of Hospitalization in Older Adults: Case Control Pre-Post Study
Source: J Med Internet Res. 2019 Oct 17;21(10):e13440. doi: 10.2196/13440 (PMC6913375; doi:10.2196/13440)
Supplement: Multimedia Appendix 1 [file jmir_v21i10e13440_app1.pdf]

APPENDIX: Avatar Protocols

Day #	Time	Brief Descriptions of Tasks
Odd	First interaction after 5 am	Depression screen & loneliness screen<br>If bed-bound: toileting check & bed exercises
Odd	9:30 am (post-breakfast)	Cognitive engagement/assessment
Odd	11 am (between breakfast/lunch)	Reorientation: weekday, nurse name, weather<br>Delirium screen
Odd	1:30 pm (post-lunch)	Clock exercise/reorientation<br>If bed-bound: comfort check
Odd	4 pm (between lunch/dinner)	Depression & delirium screen
Odd	6:30 pm (post-dinner)	If bed-bound: toileting check, comfort check<br>If not bed-bound: ambulation check
Odd	8:30 pm (evening)	Loneliness & delirium screen
Odd	Any interaction after 11 pm but before 5 am	[Encourage patient to go back to sleep, e.g. "It's ____ at night, can we please go back to sleep?"]
Even	First interaction after 5 am	Depression & delirium screen<br>If bed-bound: toileting check & bed exercises
Even	9:30 am (post-breakfast)	Reorientation: location, hospital, meal
Even	11 am (between breakfast/lunch)	Clock exercise/reorientation<br>Cognitive engagement/assessment<br>Delirium screen
Even	1:30 pm (post-lunch)	Satisfaction check
Even	4 pm (between lunch/dinner)	Loneliness & delirium screen<br>If bed-bound: comfort check<br>If not-bed-bound: ambulation check
Even	6:30 pm (post-dinner)	Clock exercise/reorientation
Even	8:30 pm (evening)	Depression & delirium screen<br>If bed-bound: toileting check, comfort check
Even	Any interaction after 11 pm but before 5 am	[Encourage patient to go back to sleep, e.g. "It's ____ at night, can we please go back to sleep?"]
