# Supplementary material for: A Protocol-Driven, Bedside Digital Conversational Agent to Support Nurse Teams and Mitigate Risks of Hospitalization in Older Adults: Case Control Pre-Post Study
Source: J Med Internet Res. 2019 Oct 17;21(10):e13440. doi: 10.2196/13440 (PMC6913375; doi:10.2196/13440)
Supplement: Multimedia Appendix 2 [file jmir_v21i10e13440_app2.pdf]

APPENDIX: Rounding Script

A. For Subjects with an Avatar

[Knock on patient door.]
Hello Mr/Ms/Mrs. _____________________, my name is ______________________________,
I am a research assistant working on the avatar project. How are you today?
I just came by to check on you. May I please sit down?
I want to make sure things are going well for you while you are in the hospital.
What do you think of your avatar? (point to tablet with avatar)
Do you need any help with the avatar or anything in your room?
Is there anything else I can do for you?
Thank you.
Either myself or another research assistant will come by to visit you again tomorrow. Goodbye.

B. For Subjects without an Avatar

[Knock on patient door.]
Hello Mr/Ms/Mrs. _____________________, my name is ______________________________,
I am a research assistant working on the avatar project. How are you today?
I just came by to check on you. May I please sit down?
I want to make sure things are going well for you while you are in the hospital.
What do you think of your room?
Do you need any help with anything in your room?
Is there anything else I can do for you?
Thank you.
Either myself or another research assistant will come by to visit you again tomorrow. Goodbye.
