# Supplementary figures and images for: A Protocol-Driven, Bedside Digital Conversational Agent to Support Nurse Teams and Mitigate Risks of Hospitalization in Older Adults: Case Control Pre-Post Study
Source: J Med Internet Res. 2019 Oct 17;21(10):e13440. doi: 10.2196/13440 (PMC6913375; doi:10.2196/13440)

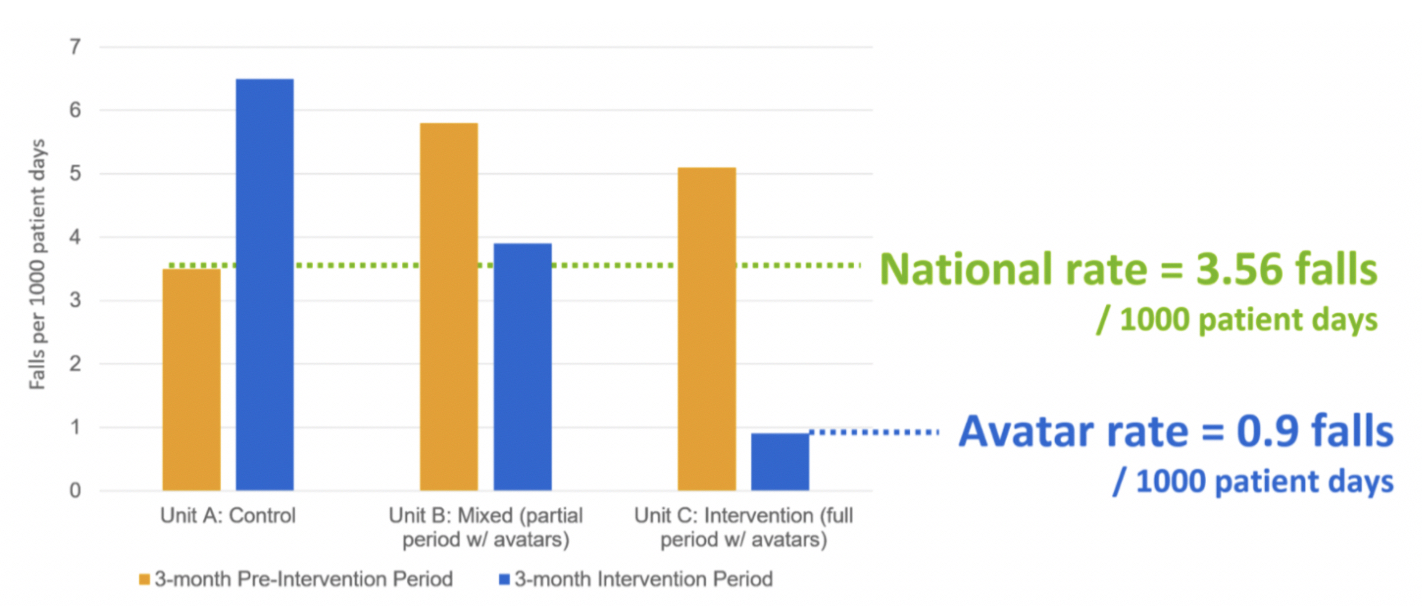

Supplement: Multimedia Appendix 3 [file jmir_v21i10e13440_app3.png]
